# The beneficial effects of inhaled nitric oxide in patients with severe traumatic brain injury complicated by acute respiratory distress syndrome: a hypothesis

**DOI:** 10.1186/1752-2897-2-1

**Published:** 2008-01-14

**Authors:** Thomas J Papadimos

**Affiliations:** 1 Department of Anesthesiology, University of Toledo, College of Medicine, 3000 Arlington Avenue, Toledo, Ohio 43614, USA

## Abstract

**Background:**

The Iraq war has vividly brought the problem of traumatic brain injury to the foreground. The costs of death and morbidity in lost wages, lost taxes, and rehabilitative costs, let alone the emotional costs, are enormous. Military personnel with traumatic brain injury and acute respiratory distress syndrome may represent a substantial problem. Each of these entities, in and of itself, may cause a massive inflammatory response. Both presenting in one patient can precipitate an overwhelming physiological scenario. Inhaled nitric oxide has recently been demonstrated to have anti-inflammatory effects beyond the pulmonary system, in addition to its ability to improve arterial oxygenation. Furthermore, it is virtually without side effects, and can easily be applied to combat casualties or to civilian casualties.

**Presentation of hypothesis:**

Use of inhaled nitric oxide in patients with severe traumatic brain injury and acute respiratory distress syndrome will show a benefit through improved physiological parameters, a decrease in biochemical markers of inflammation and brain injury, thus leading to better outcomes.

**Testing of hypothesis:**

A prospective, randomized, non-blinded clinical trial may be performed in which patients meeting the case definition could be entered into the study. The hypothesis may be confirmed by: (1) demonstrating an improvement in physiologic parameters, intracranial pressure, and brain oxygenation with inhaled nitric oxide use in severely head injured patients, and (2) demonstrating a decrease in biochemical serum markers in such patients; specifically, glial fibrillary acidic protein, inflammatory cytokines, and biomarkers of the hypothalamic-pituitary-adrenal axis, and (3) documentation of outcomes.

**Implications of hypothesis:**

Inhaled nitric oxide therapy in traumatic brain injury patients with acute respiratory distress syndrome could result in increased numbers of lives saved, decreased patient morbidity, decreased hospital costs, decreased insurance carrier and government rehabilitation costs, increased tax revenue secondary to occupational rehabilitation, and families could still have their loved ones among them.

## Background

Traumatic brain injury (TBI) affects 1.4 million Americans annually, which includes 1.1 million emergency department visits, 235,000 hospitalizations, and 50,000 deaths [1]. Approximately 5.3 million Americans are disabled with TBI [2] at a cost of $60 billion annually [3]. The Iraq war has provided additional cases and cost. At least 28% of wounded personnel have TBI resulting in $600,000 to $4,300,000 of care per patient [4-6]. This is based on 2824 wounded personnel as of August 2005 [6].

Complications occur frequently in TBI, and respiratory dysfunction represents a primary non-neurological system failure [7]. These patients are confronted with a massive inflammatory response with the release of cytokines [8] and neuropeptides [9] that are deleterious to the brain. Furthermore, this inflammatory response renders the lungs less tolerant of stressors causing ischemia-reperfusion and subsequent mechanical insults [10], i.e., massive brain injury may incite ventilator induced lung injury. This occurs through neurogenic pulmonary edema [7], ventilator associated pneumonia [11], and/or acute lung injury (ALI)/adult respiratory distress syndrome (ARDS) [12] that may be secondary to inflammatory ultrastructural changes in pneumatocyte type II cells [13] through the initiation/migration of activated neutrophils into the lungs [14]. In the face of severe pulmonary insufficiency, such as occurs in neurogenic pulmonary edema, pneumonia, and ALI/ARDS, oxygen delivery to the brain may be compromised. INO delivered at 10–80 parts per million is an effective pulmonary vasodilator that rapidly degrades in vivo [15] and improves arterial oxygenation [16-20]. However, clinical trials have not shown improved outcomes with its use in ARDS [21-23], including a large phase III study in the United States [24]. Nonetheless, inhaled nitric oxide (INO) has been successfully used twice in TBI patients with ALI/ARDS [25,26].

In severe TBI (Glasgow coma scale {GCS} ≥ 8) it has been recommended that the partial pressure of oxygen in arterial blood be maintained at a minimum of 100 mm Hg [27], cerebral perfusion pressure maintained between 60–70 mm Hg [28], and the partial pressure of carbon dioxide in arterial blood maintained at 32–35 mm Hg [29]. Increased intracranial pressure (ICP) may then be prevented from occurring. Effective oxygen delivery and decreased inflammation will assist in meeting these parameters.

Very recent basic science and clinical research has brought into question the results of the above-mentioned ARDS trials, especially as they may relate to TBI. Mathru et al have demonstrated that INO attenuates ischemia-reperfusion injury in the lower extremities of humans [30], and Gazoni et al have demonstrated such attenuation in animal lungs [31]. Hu et al concluded that INO decreased oxidative damage and inflammation along with reduced alveolar leakage in mature adult rat lungs [32]. Most importantly, Aaltoren et al have shown that pigs with meconium aspiration have hippocampal neuronal injury [33], however when INO is administered to pigs with meconium aspiration, hippocampal neuronal injury is inhibited [34]. This occurs through diminished DNA oxidation in the hippocampus and is accompanied by decreased levels of glutathione, a biomarker of oxidative stress [34]. Finally, Da et al demonstrated that INO, with concurrent administration of steroids, will decrease the inflammatory response in porcine sepsis through up-regulation of the glucocorticoid receptor (GR) [35].

Thus, use of INO in patients with severe TBI and ARDS will show a benefit through improved physiological parameters and a decrease in biochemical markers of inflammation and brain injury, leading to better outcomes.

## Presentation of the hypothesis

While INO is a potent pulmonary vasodilator, and has been thought to remain only in the pulmonary system, recent work has demonstrated that INO may go downstream to improve other organs [35] in the following manner. The view that red blood cells (RBC) consume NO has been altered to one in which the RBC is a deliverer of NO [36]. NO reacts, not only with heme iron, but also with cysteine (Cys)-93 on the hemoglobin β-unit [37]. NO reactions with heme iron cause NO's inactivation, but S-nitrosylation of Cys-93 makes hemoglobin a carrier of NO bioactivity [38]. Also, an increase in S-nitrosothiol proteins occurs in sepsis (including RBC S-nitrosothio-hemoglobin and hemoglobin [Fe]NO) [39,40]. This accumulation of hemoglobin [Fe]NO as a 5-coordinate α-heme NO does not allow NO release to the Cys-β93 residue. However, dissociation of oxygen from the 5-coordinate α-heme-NO occurs so that delivery of oxygen occurs without an extensive vasodilation [41]. Thus, according to Goldfarb and Cinel, NO excess that interacts with hemoglobin will lead to products that prevent NO toxicity [42]. Goldfarb and Cinel also point out that S-nitrosylated albumin can transport NO bioactivity downstream, i.e., to other organs [42] and that NO stabilized through hemoglobin or other proteins by reversible S-nitrosylation may be the way NO extrapulmonary effects get downstream [42].

INO and glucocorticoid regulation may be important, not only in sepsis, but also in TBI. Da et al have demonstrated that glucocorticoid receptor (GR) up-regulation decreased the inflammatory response in a porcine model of sepsis using INO in combination with glucocorticoids (neither intervention worked well alone) [35]. In contrast to Da's work, though, up-regulation of GR in the central nervous system has been considered detrimental in some animal models of TBI [43-46], but these studies did not involve INO. In humans high levels of total serum cortisol (CORT), adrenocorticotropic hormone (ACTH), and catecholamines are present early in TBI [47,48]. However, a low plasma ACTH concentration early in TBI is associated with better intensive care unit survival [49,50]. This may be part of an adaptive down-regulation as demonstrated by Lee et al in which cortical GR expression was down regulated after 6 hours of injury in the ischemic cortex of rats [51]. Thus indicating an organism's attempt at neuroprotection. It may be that INO reaching the central nervous system allows brain GR to be down regulated.

In view of new findings on its downstream effects and lack of side effects [52], INO may be delivered to the brain and cause GR expression in the brain/hippocampus to be muted. Thus enhancing a neuroprotective effect while at the same time allowing the rest of the body to up-regulate GR in response to steroids and INO administration, and assisting the body in its anti-inflammatory efforts.

## Testing the hypothesis

The hypothesis may be confirmed by achieving the following aims: (1) demonstrating an improvement in physiologic parameters, ICP, and brain oxygenation with INO use in patients with severe TBI, and (2) demonstrating a decrease in biochemical serum markers of TBI with INO use. Specifically, glial fibrillary acidic protein (GFAP, which is specific for TBI [53,54]), inflammatory cytokines (TGF-β, TNF-α, IL-2, IL-6, IL-1β), CORT, ACTH, and cortisol-binding globulin will be evaluated.

A prospective, randomized, non-blinded clinical trial may be performed in which patients meeting the following case definition could be entered into the study: a subject whose GCS is ≥ 8, who has clinically qualified for intracranial pressure monitoring, whose trachea is intubated, whose oxygenation and ventilation is being supported by a ventilator, and in whom the ratio of partial pressure of oxygen in arterial blood to inspired oxygen is less than 200 with radiographic evidence of lung injury. The subjects should be randomized into two groups, those that will receive treatment without INO, and those who will receive INO. Invasive monitoring of CNS, renal, and cardiopulmonary parameters will be necessary. Follow-up at 28 days and 6 months can be through hospital records, an information-gathering tool, and the social security death index.

Biochemical markers will be evaluated by enzyme-linked immunosorbent assays (ELISA). Physiologic monitors shall include: pulmonary artery catheter for cardio-pulmonary-vascular indices (cardiac output (CO), cardiac index (CI), mixed venous oxygenation (SVO2), central venous pressure (CVP), systemic vascular resistance index (SVRI), pulmonary vascular resistance index (PVRI), stroke volume index (SVI), right ventricular ejection fraction (RVEF), right ventricular end diastolic volume (RVEDV), left ventricular stroke work index (LVSWI), right ventricular stroke work index (RVSWI), oxygen delivery (DO2), oxygen uptake (VO2), and oxygen extraction ratio (O2ER)), arterial line for blood pressure, foley catheter with abdominal pressure monitor, LICOX^® ^Brain Oxygen tissue monitor (records brain partial pressure of oxygen, intracranial pressure, and brain temperature), cerebral oximetry, pulse oximetry, and transcranial doppler monitor for middle cerebral artery velocities. Also arterial blood gases, cerebral perfusion pressure, lactate, and methemoglobin will be monitored.

The subjects' entire physiologic/biochemical/hematologic profiles will be available for analysis, such as hemoglobin, hematocrit, electrolytes, etc., as will the injury severity score (ISS) and Apache II score.

## Implications of hypothesis

Inhaled nitric oxide in humans with TBI and ARDS has been used successfully on two occasions to improve outcomes. It has also been shown to be effective in hippocampal preservation in animals. Positive results could immediately affect treatment of military and civilian TBI patients worldwide. A decreased inflammatory response and increased arterial oxygen tension in TBI patients with ARDS, through the use of INO, could potentially lead to decreased ICP and better brain oxygenation. This would result in increased numbers of lives saved, decreased patient morbidity, decreased hospital costs, decreased insurance carrier and government rehabilitation costs, increased tax revenue secondary to occupational rehabilitation, and families could stay intact.

## Competing interests

The author(s) declare that they have no competing interests.
